# Non-intimate Relationships and Psychopathic Interpersonal and Affective Deficits as Risk Factors for Criminal Career: A Comparison Between Sex Offenders and Other Offenders

**DOI:** 10.3389/fpsyg.2021.600370

**Published:** 2021-08-24

**Authors:** Fabio Ferretti, Andrea Pozza, Fulvio Carabellese, Adriano Schimmenti, Gianluca Santoro, Gabriele Mandarelli, Giacomo Gualtieri, Felice Carabellese, Roberto Catanesi, Anna Coluccia

**Affiliations:** ^1^Department of Medical Sciences, Surgery and Neurosciences, University of Siena, Siena, Italy; ^2^Kore University of Enna, Enna, Italy; ^3^Forensic Psychiatry, University of Bari, Bari, Italy

**Keywords:** sex offenders, risk factors, psychopathy, personality traits, life events

## Abstract

Sex-offenders are at risk of criminal recidivism. For the treatment to be truly effective, it must be individualized. For this purpose, an accurate assessment should focus on criminological, psychological, and psychopathological features. The present study compared sex offenders with other offenders on historical experiences (i.e., problems with violence, anti-social behaviors, problems with personal relationships, problems with substance use, traumatic experiences, and parenting style). In addition, given the association between life events and psychopathy, we explored whether the relation between life events and crime type (sexual crime vs. other types of crime) might be moderated by psychopathy traits (interpersonal and affective deficits and antisocial behavior). Eighty-eight sex offenders (76% of whom child molesters) and 102 other offenders were included. The Historical, Clinical and Risk Management - 20 item Version 3 (HCR-20V3) and Psychopathy Checklist-Revised (PCL-R) were administered. The scores of the HCR-20V3 Historical scale items were computed to assess life events. The scores of the PCL-R factors, F1 Interpersonal affective deficits and F2 Antisocial behavior, were recorded. The presence of a history of problems with non-intimate relationships was the only significant risk factor for sexual crime compared with other crimes. Interpersonal and affective deficits provided an increased likelihood of being sex offenders as compared with other offenders when problems with non-intimate relationships were possibly/partially or certainly present.

## Introduction

The history of criminal perpetrators, the developmental pattern of their careers, and the risk factors for criminal behaviors have always been considered a central topic in criminological research.

The relationship between the traumatic experiences of the sexually abused victims and the arising of dysfunctional sexual behaviors was investigated by several scholars, with the conclusion that additional causes underlying sexual offenses must be searched and that the so-called Victim/Perpetrator Paradigm is too reductive (Burton, 2008; Rasmussen, 2012).

Psychiatric disorders play an important role as well. Schizophrenia and bipolar disorder (Carabellese et al., 2012; Pozza et al., 2019, 2020) and mood and personality disorders (Dunsieth et al., 2004; Coluccia et al., 2020) were detected in sex offenders. In their research on a sample of 1346 sex offenders, Eher et al. (2019) found that 50.1% of them suffered from personality disorders. Carabellese et al. (2012) confirmed these results, highlighting the role of schizoid, narcissistic, and avoidant personality disorders.

The relevance of psychopathy on criminal behavior and violent conduct is widely recognized in the literature, and it is generally assessed by the gold standard measure Psychopathy Checklist-Revised (PCL-R; Hare and Neumann, 2006) which covers two main factors, i.e., interpersonal and affective deficits and antisocial behavior (Carabellese et al., 2008; Leistico et al., 2008; Campbell et al., 2009; Campobasso et al., 2009; Kennealy et al., 2010; Yang et al., 2010; Ismail and Looman, 2018; Mazzoni et al., 2018). The association between psychopathy and sexual crimes emerged in several studies, including a meta-analysis conducted by Hanson et al. (2009) which found that the presence of psychopathy in sex offenders constitutes a predictive factor for criminal recidivism in sexual offenses and other types of offenses. High scores on the PCL-R were found to be associated with experiences of neglect and abuse (Kimonis et al., 2011; Schraft et al., 2013; Ometto et al., 2016; Sevecke et al., 2016). Other studies (e.g., Schimmenti et al., 2020) have shown that the relational failure of caregivers in the attachment system, due to experiences of neglect, early abandonment or loss of caregivers, can lead the child to difficulty in interpersonal self-regulation skills. These interpersonal difficulties can in turn represent a risk factor in adults for aggressive behavior, difficulties in emotion regulation and sexual problems, as it can be found in many cases of psychopathy, sadism, or paraphilia.

Such a kaleidoscope of different situations makes it difficult to identify those risk factors that can affect the dysfunctional sexual behavior of sex offenders. Due to the variety of factors that must be considered when studying sexual crimes, investigating sex offenders’ criminal career through different risk factors is a topic that still deserves the attention of researchers. Although the evidence about these relationships is still controversial, at least partly, sex offenders seem to show a higher prevalence of physical, sexual, and emotional abuse during their childhood (Lee et al., 2002; Jespersen et al., 2009; Seto and Lalumière, 2010; Drury et al., 2019) and a history of family dysfunctions (Lee et al., 2002), poor parenting style (Sigre-Leirós et al., 2016) and symptoms of social anxiety (Porter et al., 2015). In their review, Kraanen and Emmelkamp (2011) reported that sex offenders’ criminal career is associated with substance abuse, alcohol, and drug misuse.

Among the psychometric instruments for professional risk assessment, the Historical, Clinical and Risk Management - 20 item Version 3 (HCR-20 Version 3; Douglas et al., 2013) represents a psychometrically sound instrument, designed to give a comprehensive framework for the entire process of risk assessment (Douglas et al., 2013). The historical items of the HCR-20V3 provide a series of information on the subject’s existential path. The items recall some factors among those mentioned (items H1, H2, H3, H5, H8) that could have a significant role in the criminal career. Moreover, the historical items of HCR-20V3 take into consideration other factors, in particular personality disorders (H7) and major mental disorders (H6). In addition to being correlated with violent behavior (Carabellese et al., 2020a) and sexual offenses (Somma et al., 2020), such disorders can interact with the psychopathic dimension (Wong and Olver, 2016), another personality factor notoriously correlated with the risk of criminal recidivism (Hanson and Morton-Bourgon, 2005). Cartwright et al. (2018) suggested that the HCR-20V3 plays an important role in the assessment and management of sex offenders beyond the assessment of the risk of relapse. The HCR-20V3 has proved to be a valid predictor of violent conduct in perpetrators of crimes, and its use as a predictive tool has also been suggested in sex offenders (Cartwright et al., 2018). The factors that the HCR-20V3 takes into consideration, especially in the historical items, represent a valid support for a professional judgment aimed to develop a personalized therapeutic project (Yates, 2013; Gannon et al., 2019).

The aim of this study was to explore the relevance of life events in criminal outcome, comparing sex offenders with other offenders about historical experiences assessed by the HCR-20V3 related to problems with violence, anti-social behaviors, problems with personal relationships, problems with substance use, traumatic experiences, and parenting style. This is the first study which explored risk factors in sex offenders by using HCR-20V3 in its validated Italian version (Caretti et al., 2019) and one of the few studies which investigated the discriminant role of these life events comparing sex offenders (SO) with other offenders (OO). In addition, given the association between life events and psychopathy, we explored whether the relation between certain life events measured by the HCR-20 V3 and the type of crime (sexual crime vs. other types of crime) might be moderated by psychopathy traits, i.e., interpersonal and affective deficits and antisocial behavior.

## Materials and Methods

### Participants and Setting

This research is part of a national multicenter project authorized by the Penitentiary Administration Department of the Ministry of Justice. The study was reviewed and approved by the Director and ethical board of Penitentiary Administration Department of Ministry of Justice in accordance with the current ethical standards and in compliance with the rules concerning the privacy of data related to the perpetrators of sexual crimes. This multicenter study was realized with the collaborations of the penitentiary institutions of six Italian regions (Lombardia, Veneto, Toscana, Lazio, Puglia e Sicilia). The regions involved were chosen for the presence of university centers that planned the enrollment of the sample, the collection and analysis of the data. The researchers had previously been trained in the use of the tools. All the prisoners enrolled in the study received a final conviction for their crimes. Data were collected during 2015 and 2016 and only the prisoners who had previously given their consent to meet the researchers were included in the study. For legal and privacy-related reasons, data will not be made available.

The exclusion criteria were applied to both the SO and the OO groups and consisted of the lack of provision of the informed consent and the presence of a lifetime psychiatric diagnosis which was considered by the Penitentiary Administration an exclusion criterion to prevent the fact that the relation between life events and sexual crime might be attributed to a psychiatric history as psychiatric disorders are observed in sex offenders (Dunsieth et al., 2004; Carabellese et al., 2012; Eher et al., 2019). The presence of a lifetime psychiatric diagnosis was established by the penitentiary administration if the individual had received or was receiving a psychiatric treatment, i.e., psychiatric medications or psychotherapeutic treatments prescribed or delivered by a mental health professional in any period during his life.

In Italy, the number of sexual offenses leading to the internment of the offenders in the so-called High-Security Forensic Psychiatry Residences (i.e., the psychiatric facilities hosting socially dangerous offenders with a mental disorder) is relatively low (3.4%) (Catanesi et al., 2019). As for the sex offenders with mental disorders not serious enough to exclude criminal responsibility, in Italy they are sentenced in prison, but in this case, we had no data concerning them because the prisoners with psychiatric disorders were excluded from the study by the Penitentiary Administration.

Eighty-eight SO and 102 OO were assessed by researchers trained in the administration of the tools, to guarantee uniformity and homogeneity in data collection. The category SO was declined according to the definition given by Myers et al. (2005), even though the majority (76%) of these inmates were child molesters. The final sample of 88 sex offenders did not commit any other crimes than sexual offenses, possibly in relapse. The data concerning the fact that the SOs had been convicted for the first time or they were recidivists, though, was kept confidential by the prison management. It must also be said that SOs in Italy are either detained in separate prisons (as happens for example in Puglia), or they are placed in special sections within the individual prisons.

The category OO included a heterogeneous set of crimes, such as personal crimes (homicide, assault), property crimes (robbery, fraud), and crimes against the State, but not sex offenses.

A preliminary meeting with prisoners and social workers was scheduled before the assessment, to present the research project and collect participants’ written consent (Mandarelli et al., 2017). At this stage, about 18% of prisoners refused to give informed consent and participate. However, the researchers did not know the number of those who had initially refused to participate to the preliminary meeting in which the project was presented. In fact, the request as to whether the prisoners intended to participate to the preliminary meeting had been made by the penitentiary institutions and kept confidential. The various stages of sample recruitment are shown in Supplementary Material.

### Measures

The Psychopathy Checklist-Revised (PCL-R; Hare and Neumann, 2006) in its validated Italian version (Caretti et al., 2011) was administered to assess the presence of psychopathy. PCL-R factor 1 captures traits dealing with the interpersonal and affective deficits of psychopathy (e.g., shallow affect, superficial charm, manipulativeness, and lack of empathy), whereas factor 2 deals with symptoms relating to antisocial behavior (e.g., criminal versatility, impulsiveness, irresponsibility, poor behavior controls, and juvenile delinquency) (Hare et al., 1989). The scores of the two different components of factors F1 and F2 were recorded, as well as the values of the different components within factors F1 and F2. A threshold score of PCL-R equal to or greater than 25 was established to identify the condition of psychopathy, as indicated in studies conducted on European populations (Grann et al., 1998; Andersen et al., 1999; Jüriloo et al., 2013).

The HCR-20V3 Italian version (Douglas et al., 2013; Caretti et al., 2019), a tool assessing the risk of violence, was administered. Some items of the Historical Scale were considered to analyze the existence of lifetime problematic experiences that can be considered risk factors for the development of sexually based crimes. According to the literature about risk factors for sex offending, the following items were selected among those belonging to the Historical Scale: (a) H1 – problems with violence; (b) H2 – problems with other antisocial behavior; (c) H3 – problems with relationships; (d) H5 – problems with substance use; (e) H8 – problems with traumatic experiences. Items H1 and H2 are divided in sub-items expressing three age classes: (1) child, 12 years and younger; (2) adolescent, 13–17 years; (3) adult, 18 years and older. The item H3 is divided in two sub-items (intimate and non-intimate relationships), and the item H8 is also divided in two sub-items (victimization/trauma and adverse child rearing experiences). Every item is scored 0, 1, or 2: 0 if the item is definitely absent, 1 if the item possibly is present, or present to a minor/moderate degree, and 2 if the item is definitely present. All the researchers involved had been trained through role-playing and internships to administer the tools before the research was initiated.

Together with the assessment scales, the anamnestic and criminological data were collected through the examination of the personal data sheets.

### Statistical Analysis

Descriptive statistics were used to describe subjects’ characteristics (mean and standard deviation for continuous measures, frequencies, and relative frequencies for categorical variables). In accordance with the manual of the instrument (Caretti et al., 2019), the presence of the risk factors was measured by the HCR-20V3 Historical item scores which were coded as a categorical variable (i.e., response categories: “No” vs. “Possibly/Partially” vs. “Yes”).

The characteristics of incarcerated groups (SO vs. OO) and their risk factors assessed with HCR-20V3 were compared using parametric tests for the continuous variables (Student’s *t-*test, once verified the assumption of normality with Kolmogorov-Smirnov test) and non-parametric tests for categorical variables (Chi-square and Fisher’s Exact Test).

A binary logistic model was used to estimate the effect of the HCR-20V3 historical risk factors in predicting criminal careers, with being SO or OO as the dichotomous dependent variable. Only those risk factors that showed a significant difference in the comparison of the two groups were included as predictors in the regression model. The assumptions related to the sample size adequacy, the independence of observations, and the lack of multicollinearity among the independent variables were verified. Model fit was assessed through Hosmer-Lemeshow test. The Wald’s statistic was used to analyze the predictors’ contribution to the explanation of the dependent variable. The Odds Ratio and their 95% confidence intervals were computed for each predictor’s category. A second binary logistic model was fitted to assess the interaction effects with PCL-Rs’ Factors 1 and 2.

Data analysis was performed with SPSS-IBM v25 software, setting significance at *p* < 0.05.

## Results

Table 1 summarizes the characteristics of the two groups of subjects (SO vs. OO). The total mean age was 45.12 ± 12.732, and it was significantly higher in the SO group (*t*_(__188__)_ = −3.947; *p* = 0.000). About half of the convicted was unemployed (46.8%), and a percentage of 11.1% showed a diagnosis of psychopathy (PCL-R score ≥ 25). No association was found between the employment status, or the diagnosis of psychopathy, and the two groups of subjects.

**TABLE 1:** “Sex Offenders” (SO) and “Other Offenders” (OO) characteristics: age, employment status, educational attainment, and diagnosis of psychopathy.

		Total	SO (*n* = 88)	OO (*n* = 102)	Statistic	*p*
Age (mean ± SD)		45.12 ± 12.732	48.90 ± 13.281	41.85 ± 11.232	*t*_(__188__)_ = −3.947	**0.000**
Employment status *n*.(%)	*Employed*	101 (53.2)	50 (56.8)	51 (50.0)	Fisher’s Exact Test	0.383
	*Unemployed*	89 (46.8)	38 (43.2)	51 (50.0)		
Educational attainment *n*.(%)	*Primary school*	13 (6.8)	2 (2.3)	11 (10.8)	χ^2^_(__3__)_ = 24.186	**0.000**
	*Secondary school*	78 (41.1)	26 (29.5)	52 (51.0)		
	*High school*	73 (38.4)	39 (44.3)	34 (33.3)		
	*University degree*	26 (13.7)	21 (23.9)	5 (4.9)		
Diagnosis of psychopathy (PCL-R ≥ 25) *n*.(%)	*Yes*	21 (11.1)	9 (10.2)	12 (11.8)	Fisher’s Exact Test	0.819
	*No*	169 (88.9)	79 (89.8)	90 (88.2)		

*Bold values represent statistically significant *p*-values.*

Considering the educational attainment, the secondary school was the most frequent (41.1%), followed by high school (38.4%). A higher school level was associated with the SO group (χ^2^_(__3__)_ = 24.186; *p* = 0.000). Chi-Square *post hoc* tests were performed by checking the significance of the adjusted residuals of the contingency table cells, with Bonferroni correction. The results of the *post hoc* tests showed that SO and OO groups showed significant adjusted residuals for secondary school and university degree (both, respectively: *p* = 0.022 and *p* = 0.001).

The results of the comparisons on the HCR-20 V3 between SO and OO on the risk factors carried out by non-parametric tests are presented in Table 2. *Post hoc* analysis results are displayed in the note of Table 3. Some of the risk factors assessed as definitely present (coded as “*Yes*”) showed a high prevalence in the total group of subjects. A history of problems with violence as an adult (18 and over) was detected in the 62.1% of subjects, and problems with antisocial behavior, once again as an adult, pertained to the 46.3% of the prisoners enrolled in the study. Problems with relationships showed a high prevalence in this sample, intimate and non-intimate as well (respectively: 47.9 and 35.8%). A history of problems with substance use was definitely found in the 34.2% of the subjects, whilst risk factors related to traumatic experiences were detected in a lower proportion: 30.0% experienced a victimization or trauma, and 26.8% reported experiences of adverse child rearing.

**TABLE 2:** Comparison among “sex offenders” (SO) and “other offenders” (OO) about the risk factors assessed with HCR-20-historical scale (items: H1a, H1b, H1c, H2a, H2b, H2c, H3a, H3b, H5, H8a, H8b).

		Total	SO (*n* = 88)	OO (*n* = 102)	Statistic	*p*
HCR-20V3 H1a Violence: as a child (12 and under) *n*.(%)	*No*	144 (75.8)	71 (80.7)	73 (71.6)	χ^2^_(__2__)_ = 5.436	0.066
	*Possibly/Partially*	32 (16.8)	9 (10.2)	23 (22.5)		
	*Yes*	14 (7.4)	8 (9.1)	6 (5.9)		
HCR-20V3 H1b Violence: as an adolescent (13–17) *n*.(%)	*No*	125 (65.8)	71 (80.7)	54 (52.9)	χ^2^_(__2__)_ = 19.285	**0.000**
	*Possibly/Partially*	40 (21.1)	7 (8.0)	33 (32.4)		
	*Yes*	25 (13.2)	10 (11.4)	15 (14.7)		
HCR-20V3 H1c Violence: as an adult (18 and over) *n*.(%)	*No*	30 (15.8)	18 (20.5)	12 (11.8)	χ^2^_(__2__)_ = 7.960	**0.019**
	*Possibly/Partially*	42 (22.1)	12 (13.6)	30 (29.4)		
	*Yes*	118 (62.1)	58 (65.9)	60 (58.8)		
HCR-20V3 H2a Other antisocial behavior: as a child (12 and under) *n*.(%)	*No*	151 (79.5)	72 (81.8)	79 (77.5)	χ^2^_(__2__)_ = 1.882	0.390
	*Possibly/Partially*	19 (10.0)	6 (6.8)	13 (12.7)		
	*Yes*	20 (10.5)	10 (11.4)	10 (9.8)		
HCR-20V3 H2b Other antisocial behavior: as an adolescent (13–17) *n*.(%)	*No*	108 (56.8)	64 (72.7)	44 (43.1)	χ^2^_(__2__)_ = 17.288	**0.000**
	*Possibly/Partially*	46 (24.2)	12 (13.6)	34 (33.3)		
	*Yes*	36 (18.9)	12 (13.6)	24 (23.5)		
HCR-20V3 H2c Other antisocial behavior: as an adult (18 and over) *n*.(%)	*No*	67 (35.3)	44 (50.0)	23 (22.5)	χ^2^_(__2__)_ = 16.615	**0.000**
	*Possibly/Partially*	35 (18.4)	10 (11.4)	25 (24.5)		
	*Yes*	88 (46.3)	34 (38.6)	54 (52.9)		
HCR-20V3 H3a Relationships: intimate *n*.(%)	*No*	43 (22.6)	19 (21.6)	24 (23.5)	χ^2^_(__2__)_ = 0.293	0.864
	*Possibly/Partially*	56 (29.5)	25 (28.4)	31 (30.4)		
	*Yes*	91 (47.9)	44 (50.0)	47 (46.1)		
HCR-20V3 H3b Relationships: non-intimate *n*.(%)	*No*	68 (35.8)	33 (37.5)	35 (34.3)	χ^2^_(__2__)_ = 7.416	**0.025**
	*Possibly/Partially*	54 (28.4)	17 (19.3)	37 (36.3)		
	*Yes*	68 (35.8)	38 (43.2)	30 (29.4)		
HCR-20V3 H5 Substance use *n*.(%)	*No*	88 (46.3)	55 (62.5)	33 (32.4)	χ^2^_(__2__)_ = 25.697	**0.000**
	*Possibly/Partially*	37 (19.5)	19 (21.6)	18 (17.6)		
	*Yes*	65 (34.2)	14 (15.9)	51 (50.0)		
HCR-20V3 H8a Traumatic experiences: victimization/trauma *n*.(%)	*No*	97 (51.1)	50 (56.8)	47 (46.1)	χ^2^_(__2__)_ = 2.272	0.321
	*Possibly/Partially*	36 (18.9)	14 (15.9)	22 (21.6)		
	*Yes*	57 (30.0)	24 (27.3)	33 (32.4)		
HCR-20V3 H8b Traumatic experiences: adverse child rearing experiences *n*.(%)	*No*	82 (43.2)	44 (50.0)	38 (37.3)	χ^2^_(__2__)_ = 3.866	0.145
	*Possibly/Partially*	57 (30.0)	21 (23.9)	36 (35.3)		
	*Yes*	51 (26.8)	23 (26.1)	28 (27.5)		

**Post hoc* tests and the significance of the standardized adjusted residuals (ns = not significant).*

*a: No-SO: *p* = 0.000; No-OO: *p* = 0.000; Possibly/Partially-SO: *p* = 0,000; Possibly/Partially-OO: *p* = 0,000; Yes-SO = ns; Yes-OO = ns.*

*b: No-SO: *p* = ns; No-OO: *p* = ns; Possibly/Partially-SO: *p* = ns; Possibly/Partially-OO: *p* = ns; Yes-SO: *p* = ns; Yes-OO: *p* = ns.*

*c: No-SO: *p* = 0.000; No-OO: *p* = 0.000; Possibly/Partially-SO: *p* = 0,008; Possibly/Partially-OO: *p* = 0,000; Yes-SO: *p* = ns; Yes-OO: *p* = ns.*

*d: No-SO: *p* = 0.001; No-OO: *p* = 0.001; Possibly/Partially-SO: *p* = ns; Possibly/Partially-OO: *p* = ns; Yes-SO: *p* = ns; Yes-OO: *p* = ns.*

*e: No-SO: *p* = ns; No-OO: *p* = ns; Possibly/Partially-SO: *p* = ns; Possibly/Partially-OO: *p* = ns; Yes-SO: *p* = ns; Yes-OO: *p* = ns.*

*f: No-SO: *p* = 0.000; No-OO: *p* = 0.000; Possibly/Partially-SO: *p* = ns; Possibly/Partially-OO: *p* = ns; Yes-SO: *p* = 0.000; Yes-OO: *p* = 0.000.*

*Bold values represent statistically significant *p*-values.*

**TABLE 3:** Results of the binary logistic model fitting: predictive effects of HCR-20V3 Historical items.

Predictors	β	S.E.	Wald’s statistic	*p*	OR	OR 95% CI	
						Lower limit	Upper limit
**HCR-20V3 H1b Violence: as an adolescent (13–17)**							
Violence: (13–17) - *No*			Wald’s χ^2^_(__2__)_ = 5.100	0.078			
Violence: (13–17) - *Possibly/Partially*	-1.237	0,548	Wald’s χ^2^_(__1__)_ = 5.090	**0.024**	0.290	0.099	0.850
Violence: (13–17) - *Yes*	-0.389	0.617	Wald’s χ^2^_(__1__)_ = 0.398	0.528	0.678	0.202	2.270
**HCR-20V3 H1c Violence: as an adult (18 and over)**							
Violence: (18 and over) - *No*			Wald’s χ^2^_(__2__)_ = 2.953	0.228			
Violence: (18 and over) - *Possibly/Partially*	-0.508	0.608	Wald’s χ^2^_(__1__)_ = 0.700	0.403	0.602	0.183	1.979
Violence: (18 and over) - *Yes*	0.330	0.495	Wald’s χ^2^_(__1__)_ = 0.445	0.505	1.391	0.527	3.672
**HCR-20V3 H2b Other antisocial behavior: as an adolescent (13–17)**							
Other antisocial behavior: (13–17) - *No*			Wald’s χ^2^_(__2__)_ = 0.756	0.685			
Other antisocial behavior: (13–17) - *Possibly/Partially*	-0.480	0.579	Wald’s χ^2^_(__1__)_ = 0.687	0.407	0.619	0.199	1.926
Other antisocial behavior: (13–17) - *Yes*	-0.160	0.694	Wald’s χ^2^_(__1__)_ = 0.053	0.817	0.852	0.218	3.323
**HCR-20V3 H2c Other antisocial behavior: as an adult (18 and over)**							
Other antisocial behavior: (18 and over) - *No*			Wald’s χ^2^_(__2__)_ = 3.669	0.160			
Other antisocial behavior: (18 and over) - *Possibly/Partially*	-0.948	0.567	Wald’s χ^2^_(__1__)_ = 2.793	0.095	0.388	0.128	1.178
Other antisocial behavior: (18 and over) - *Yes*	-0.840	0.537	Wald’s χ^2^_(__1__)_ = 2.448	0.118	0.432	0.151	1.236
**HCR-20V3 H3b Non-intimate relationships**							
Relationships: non-intimate - *No*			Wald’s χ^2^_(__2__)_ = 8.127	**0.017**			
Relationships: non-intimate - *Possibly/Partially*	0.005	0.47	Wald’s χ^2^_(__1__)_ = 0.000	0.991	1.005	0.400	2.524
Relationships: non-intimate - *Yes*	1.284	0.51	Wald’s χ^2^_(__1__)_ = 6.325	**0.012**	3.610	1.327	9.819
**HCR-20V3 H5 Substance use**							
Substance use - *No*			Wald’s χ^2^_(__2__)_ = 14.402	**0.001**			
Substance use - *Possibly/Partially*	0.351	0.515	Wald’s χ^2^_(__1__)_ = 0.465	0.495	1.421	0.518	3.898
Substance use - *Yes*	-1.615	0.485	Wald’s χ^2^_(__1__)_ = 11.092	**0.001**	0.199	0.077	0.515

*Dichotomous dependent variable: type of group (SO vs. OO).*

*Abbreviations: HCR-20 V3 = Historical, Clinical and Risk Management, OO = Other offenders, OR = Odds Ratio, SO = Sex Offenders.*

*Bold values represent statistically significant *p*-values.*

Six out of eleven risk factors considered in the analysis displayed a significant association with the groups: (a,b) problems with violence (as a child and as an adult), (c,d) problems with other antisocial behavior (as a child and as an adult), (e) problems with non-intimate relationships and (f) problems with substance use. Prisoners included in the OO group seemed to be portrayed by a history of problems with violence. Considering the subscale “as an adolescent (13–17)” and summing the relative frequencies of the two categories “Possibly/Partially” or “Yes”, this risk factor was observed in the 47.1% of the OO group, against the 19.4% of the SO group (χ^2^_(__2__)_ = 19.285; *p* = 0.000). Although a history of problems with violence as an adult was more frequent in the SO group than in the OO group (respectively: 65.9 and 58.8%), the lack of this risk factor proved to be double than that of the OO group (respectively: 20.5 and 11.8%; χ^2^_(__2__)_ = 7.960; *p* = 0.019). Also the other antisocial behaviors characterized the OO group: during the adolescence, this problem was detected (“Possibly/Partially” or “Yes”) in the 56.8% of the OO group (*SO* = 27.2%; χ^2^_(__2__)_ = 17.288; *p* = 0.000), and, as an adult, this percentage was 77.4% (*SO* = 50.0%; χ^2^_(__2__)_ = 16.615; *p* = 0.000). The last risk factor associated with the OO group concerned substance use (χ^2^_(__2__)_ = 25.697; *p* = 0.000).

Finally, a history of problems with non-intimate relationships was the only risk factor linked to the criminal career of the SO: in this group, it was definitely assessed in the 43.2% of the subjects, against the 29.4% of the OO group (χ^2^_(__2__)_ = 7.416; *p* = 0.025).

The results of Student’s *t-*tests showed that as compared with OO, the group of SO had significantly higher and lower scores, respectively, on the PCL-R F1 Interpersonal and Affective Deficits and PCL-R F2 Antisocial Behavior. In both groups, the scores on the PCL-R factors were moderately correlated to each other. The results of the comparisons on the PCL-R factor scores and the correlations in the two groups are presented in Supplementary Material.

These six mentioned factors presenting a significant association with the study groups were included as predictors in a binary logistic regression model. The results of the logistic regression analysis are presented in Table 3 where the role of HCR-20V3 historical risk factors is entered as predictors and the criminal career (defined as being a SO or OO) is entered as dichotomous outcome. The lack of significance of the Hosmer-Lemeshow test (χ^2^_(__8__)_ = 7.078; *p* = 0.528) proved the goodness of the model fitting. The percentage of predicted cases was 73% out of the total group.

Three risk factors did not show a significant contribution to the dependent variable: problems with violence (as an adult) and problems with antisocial behavior (as an adolescent and as an adult).

Two risk factors provided a significant effect on the dependent variable but in favor of the “other offenders” criminal career: the possible, or partial, presence of problems with violence as an adolescent (β = −1.237; Wald’s χ^2^_(__1__)_ = 5.090; *p* = 0.024) and the certain history of substance use (β = −1.615; Wald’s χ^2^_(__1__)_ = 11.092; *p* < 0.001). According to these results, the detection of these risk factors was associated with a lower likelihood of being “SO”.

The presence of a history of problems with non-intimate relationships was the only significant risk factor explaining the outcome “SO”. The high value of the Wald’s statistic (6.325) expressed its relevant contribution to the regression model. Looking at the odds ratio, the prisoners experiencing problems with non-intimate relationships were 3.610 (95% CI 1.327–9.819) times more likely to be a “SO” than another type of offender.

Although no significant difference was found between SO and OO on their psychopathological conditions, a second logistic regression was estimated, using the only significant predictor for the SO category in interaction with the two PCL-R factors: (F1) interpersonal and affective deficits, (F2) antisocial behavior. The results of the logistic regression analysis are shown in Table 4 where the interaction effects between the significant risk factor (i.e., Non-Intimate Relationships) and the two PCL-R factors (i.e., Interpersonal and Affective Deficits and Antisocial Behavior, respectively) were included as predictors and the sexual career (i.e., being a SO vs. OO) is included as dichotomous outcome. The lack of significance of the Hosmer-Lemeshow test (χ^2^_(__6__)_ = 2.896; *p* = 0.822) confirmed the goodness of fit, even if the percentage of predicted cases was quite low (66% out of the total group). In other words, a 29% of the variance between the SO and OO groups was attributed to the psychopathy factors when we considered problems with non-intimate relationships.

**TABLE 4:** Results of the binary logistic model with SO/OO as outcome and the HCR20-V3 item H8 non-intimate as predictor, in interaction with the PCL-R Factor 1 Interpersonal and affective deficits and Factor 2 Antisocial behavior; Odds Ratio and 95% confidence interval of the Odds Ratio.

Predictors	β	S.E.	Wald’s statistic	*P*	OR	OR 95% CI	
						Lower limit	Upper limit
**Interaction effects between HCR-20V3 H3b non-intimate relationships and PCL-R factor 1**							
Non-intimate relationships: No ^∗^ PCL-R F1 Interpersonal and Affective Deficits interaction			Wald’s χ^2^_(__2__)_ = 19.766	**0.000**			
Non-intimate relationships: Possibly/Partially ^∗^ PCL-R F1 Interpersonal and Affective Deficits interaction	0.358	0.116	Wald’s χ^2^_(__1__)_ = 9.493	**0.002**	1.430	1.139	1.796
Non-intimate relationships: Yes ^∗^ PCL-R F1 Interpersonal and Affective Deficits interaction	0.251	0.076	Wald’s χ^2^_(__1__)_ = 10.824	**0.001**	1.286	1.107	1.493
**Interaction effects between HCR-20V3 H3b non-intimate relationships and PCL-R factor 2**							
Non-intimate relationships: No ^∗^ PCL-R F2 Antisocial Behavior interaction			Wald’s χ^2^_(__2__)_ = 23.521	**0.000**			
Non-intimate relationships: Possibly/Partially ^∗^ PCL-R F2 Antisocial Behavior interaction	−0.533	0.146	Wald’s χ^2^_(__1__)_ = 13.396	**0.000**	0.587	0.441	0.781
Non-intimate relationships: Yes ^∗^ PCL-R F2 Antisocial Behavior interaction	−0.231	0.072	Wald’s χ^2^_(__1__)_ = 10.291	**0.001**	0.794	0.689	0.914

*Abbreviations. HCR-20V3 = Historical, Clinical and Risk Management PCL-R = Psychopathy Checklist-Revised, SO = Sexual offenders, OO = Other offenders, OR = Odds ratio. Bold values represent statistically significant *p*-values.*

Both PCL-R factors showed a significant interaction with the existence of problems with the non-intimate relationships detected with HCR-20 V3, but with opposite effects. Increasing values of the PCL-R Factor 1 (interpersonal and affective deficits) provided an increased likelihood of being SO when problems with the non-intimate relationships were possibly/partially or certainly present (“Possibly/partially”: β = 0.358, Wald’s χ^2^_(__1__)_ = 9.493, *p* = 0.002; “Yes”: β = 0.251, Wald’s χ^2^_(__1__)_ = 10.824, *p* = 0.001). On the contrary, increasing values of the PCL-R Factor 2 (antisocial behavior) were associated to a decreased likelihood of being SO when problems with the non-intimate relationships were possibly/partially or certainly present (“Possibly/partially”: β = −0.533, Wald’s χ^2^_(__1__)_ = 13.396, *p* = 0.000; “Yes”: β = −0.231, Wald’s χ^2^_(__1__)_ = 10.291, *p* = 0.001).

## Discussion

The challenge of this study was to border the investigation concerning the association between risk factors and criminal career, to the identification of those lifetime events that can discriminate sex offenders from other offenders. The risk factors were assessed by a set of items of the HCR-20 V3 Historical scale. As compared with the SO, the OO group showed a greater likelihood of having a history of problems with violence and antisocial behavior (as adolescents and adults), together with problems with substance abuse. These findings were in line with the literature evidence. Only one risk factor, i.e., the presence of problems in non-intimate relationships, discriminated the criminal career of sex offenders from other types of criminals. In the HCR-20 V3 manual, non-intimate relationships are depicted as the bonds with the family members, the friends, or with generic acquaintances which do not involve the sexual dimension. Social isolation, emotional distance, instability or conflict, manipulation of others, inappropriate sexualization and violence in non-intimate relationships and escalation of problems are indicators of the presence of such problems. As compared with the OO group, SO showed higher and lower levels, respectively, of psychopathic interpersonal and affective deficits and antisocial behavior. Finally, the present findings confirmed the importance of deficient non-intimate relationships in predicting the criminal career of sex offenders.

Furthermore, this association was boosted by psychopathic traits related to interpersonal and affective deficits, but not by antisocial behavior. The moderator role of interpersonal/affective deficits in the relation between problems in non-intimate relationships and the risk of being SO vs. OO seems to be partially in line with a meta-analysis which indicates that this psychopathy facet is associated with violence (Kennealy et al., 2010). The present findings suggest that during clinical practice the assessment of SO should focus on social isolation, emotional distance, instability or conflict, manipulation of others, inappropriate sexualization and violence, particularly in non-intimate relationships. Tailored treatment programs aimed to prevent relapse in SO (Carabellese et al., 2020b; Gualtieri et al., 2020b) should address these deficits in non-intimate relationships and target psychopathic traits, specifically interpersonal and affective traits. Cognitive behavioral therapy has been found to be an effective treatment for psychopathic traits (Salekin et al., 2010). The fact that SO showed specific interpersonal deficits in the domain of non-intimate relationships suggests that a group format of treatment might be more helpful in addressing such difficulties in this population. In a group setting, the process of confronting with the experiences of other offenders might improve emotional awareness and regulation (Jennings and Sawyer, 2003; Levenson et al., 2009), and it might reduce the risk of drop-out from treatment, like for other clinical populations with impulsive traits (Pozza and Dèttore, 2017).

The result showing that SO had higher and lower levels of, respectively, interpersonal and affective deficits and antisocial behavior suggests that psychopathic traits could be differentially related to sex crimes and other types of crimes. This result appears consistent with literature data which extensively showed that sex offenders would be characterized by severe deficits in interpersonal empathy and emotion regulation (Gillespie et al., 2018; Schuler et al., 2021), while other types of offenders would show antisocial personality traits (Mazzoni et al., 2018; Azevedo et al., 2020).

The greater likelihood of a history of violence and antisocial behavior (as adolescents and adults), together with problems with substance abuse amongst OO as compared with SO, is a result in line with the literature evidence (e.g., Gottfredson et al., 2008). The importance of violence during the adolescence as predictor of offending patterns in adulthood is still debated (Cardwell and Piquero, 2018), even if violent behaviors during the childhood, for example the presence of aggressions (Juon et al., 2006), seem to predict serious offending in adulthood. The link between drug use and criminal behavior has received attention from scholars (Tonry and Wilson, 1990; Bennett et al., 2008; Gottfredson et al., 2008; Liu et al., 2018).

Although several studies related to sex offenders’ criminal career provided evidence of the effects of traumatic experiences during their childhood, also defined as Adverse Childhood Experiences (ACE), this relationship was not confirmed by the findings illustrated in this paper. Considering the studies which compare male sex offenders with the general population (Levenson et al., 2016; Kingston et al., 2017), sex offenders showed a larger odd of being victims of sexual abuse, verbal or physical abuse and emotional neglect, but also signals of adverse child-rearing experiences (incarcerated family members, parental dysfunctions, disordered familiar environment). Victimization or other traumatic experiences during the childhood, like physical, sexual and emotional abuse, were more prevalent in sex offenders than in other kinds of offenders (Lee et al., 2002; Jespersen et al., 2009; Seto and Lalumière, 2010; Reavis et al., 2013; Drury et al., 2019; Gualtieri et al., 2020a). In addition, evidence of adverse child-rearing experiences was more frequent as well, like a history of family dysfunctions or a condition of poor parenting style (Lee et al., 2002; Reavis et al., 2013; Sigre-Leirós et al., 2016).

Many reasons can explain the divergent findings reported in this paper. First, the methodological issues that affect this research topic. The review of McMillan et al. (2008), specifically carried out on sexual offenders against children, pointed out a series of problems related to the temporal relationship between a putative risk factor and sexual perpetration, the effects of other variables, and the complexity in modeling developmental phenomena.

Secondarily, this lack of evidence may be due to the measurement scale administered to the participants, in particular the HCR-20 V3 used to assess the risk factors. The Historical Clinical Risk Management-20 (HCRS-20) has been criticized for its low field validity in clinical forensic psychiatry settings (Jeandarme et al., 2017; Tully, 2017). On the contrary, other studies supported the psychometric validity and the clinical utility of the scale (Judges et al., 2016), highlighting that the scoring subjectivity of the scale did not impair its reliability, above all for the HCR-20 V3 historical scale (Rufino et al., 2011).

A third problem can be associated to the method used by this scale to assess the risk factors, which are described by a wide variety of lifetime events such as trauma or victimization related to parental and non-parental sexual abuse during childhood/adolescence, witnessing of domestic violence, bullying, or lifetime interpersonal victimization. As much heterogeneity of events is also listed to measure the presence of adverse child-rearing experiences (overly rigid parenting styles, unstable family relations, conviction of parents before the subject reached 10 years of age, death of a parent during childhood or adolescence, separation from parents before 17 years of age, parental substance abuse). Probably, this wide set of experiences describing a single indicator can reduce its specificity in detecting the risk factors.

Some limitations of the study should be pointed out. Firstly, the recruitment of the sample might be subjected to a bias as this process took place in agreement with the Penitentiary Administration of the Ministry of Justice. The first stage of recruitment was carried out exclusively by the prison administration, which asked the prisoners if they were interested in participating to a preliminary meeting with the researchers. On that occasion, the researchers presented the project in detail and asked the prisoners for the informed consent. However, other information that could have been useful for the purpose of the research was not provided to the researchers by the prison administration. In particular, no data were provided about the specific types of crime of the OO group or how many of both the groups of offenders were recidivists. However, it was well-established that those prisoners belonging to the group of SO did not commit any other crimes than sexual ones.

Secondly, the cross-sectional design did not allow us to draw firm conclusion about the role of the risk factors in the likelihood of being SO or OO, particularly in the risk of recidivism. Future research should use a longitudinal design to ascertain problems in non-intimate relationships and whether interpersonal/affective deficits can predict a higher risk of recidivism in sexual crime as compared with the risk of recidivism in other types of crime.

Another relevant limitation concerns the fact that individuals with a psychiatric history were excluded. This aspect reduced the external validity of this research. The findings observed in the present sample may be applied to a minority of the SO population. In fact, SO with no other criminal record and no lifetime psychiatric diagnosis typically represent only a small proportion of SO that is lower than 20%, as the lack of psychiatric comorbidities is generally observed in a 7–15% proportion of the SO population (e.g., Raymond et al., 1999; Dunsieth et al., 2004). Future studies should also include individuals with lifetime psychiatric disorders and control the effect of the presence of psychiatric conditions in the risk of being SO or OO, particularly severe psychiatric disorders such as psychosis/bipolar and personality disorders (Van Wijk et al., 2007; Coluccia et al., 2015; Chen et al., 2016; Eastman et al., 2019). However, the role of psychiatric disorders in the risk of violence has been questioned as a dimensional approach is considered more informative (Graz et al., 2009). Finally, an area that deserves further exploration regards the moderator effect of protective factors in the relation between life events and crime type. It would be interesting to examine whether certain protective factors such as the capacity to forgive oneself (Barcaccia et al., 2019) could protect the individual from the criminal behavior.

## Conclusion

The role of life events as risk factors for sexual crime is a complex and long-debated topic in criminological research. The present study is the first contribution which compared SO and OO on the risk factors assessed by the HCR-20V3 and explored the role of psychopathic traits as moderators of the relation between specific life events and the risk of having committed sexual crime or other types of crime. The present findings shed further light on this issue showing the role of deficient non-intimate relationships in predicting the criminal career of sex offenders, and suggesting that specific psychopathic traits, i.e., interpersonal/affective deficits, but not antisocial behavior, can moderate the relation between a history of problems in non-intimate relationships and sexual crime.

## Data Availability Statement

The original contributions presented in the study are included in the article/Supplementary Material, further inquiries can be directed to the corresponding author.

## Ethics Statement

The studies involving human participants were reviewed and approved by Director of Penitentiary Administration Department of Ministry of Justice. The patients/participants provided their written informed consent to participate in this study.

## Author Contributions

FF performed the statistical analysis. AP interpreted the results of the analyses and wrote the manuscript with support from FF, FuC, and FeC. AS and GS took care of the bibliographic research. FuC was the corresponding author. GM collected the data. GG contributed to the bibliographic research. FeC conceived of the presented idea and collected the data. RC and AC performed a general review. All authors contributed to the article and approved the submitted version.

## Conflict of Interest

The authors declare that the research was conducted in the absence of any commercial or financial relationships that could be construed as a potential conflict of interest.

## Publisher’s Note

All claims expressed in this article are solely those of the authors and do not necessarily represent those of their affiliated organizations, or those of the publisher, the editors and the reviewers. Any product that may be evaluated in this article, or claim that may be made by its manufacturer, is not guaranteed or endorsed by the publisher.
