# Supplementary material for: Non-intimate Relationships and Psychopathic Interpersonal and Affective Deficits as Risk Factors for Criminal Career: A Comparison Between Sex Offenders and Other Offenders
Source: Front Psychol. 2021 Aug 24;12:600370. doi: 10.3389/fpsyg.2021.600370 (PMC8421521; doi:10.3389/fpsyg.2021.600370)
Supplement: Supplementary file 1 [file Data_Sheet_1.docx]

| SAMPLE TO BE INVOLVED IN THE STUDY:  1. SO (Sex Offenders): convicted prisoners who committed sexual crimes, but no other types of crime*  2. OO (Other Offenders): inmates convicted of any type of crime, except of a sexual crime* | | | | |
| --- | --- | --- | --- | --- |
| FIRST STEP | SECOND STEP | THIRD STEP | FOURTH STEP | FIFTH STEP |
| Penitentiary institutions of six Italian regions (Lombardia, Veneto, Toscana, Lazio, Puglia, and Sicilia) asked the prisoners if they agreed to meet the researchers in a preliminary meeting for the presentation of the project | Researchers presented the project to prisoners who had agreed to participate in the preliminary meeting | At the end of the presentation of the project, the researchers asked the prisoners to sign the informed consent necessary to be involved in the study | About 18% of the prisoners who attended the preliminary meeting did not give informed consent to be involved in the study ** | 88 sex offenders and 102 other offenders gave informed consent to be involved in the study and were assessed by researchers trained for the administration of the tools (HCR-20 and PCL-R) |

**Supplementary material 1**. Process of recruitment and sampling.

*The first exclusion criterion was applied at this stage: prisoners with a lifetime psychiatric diagnosis were excluded by the Penitentiary institutions.

**The second exclusion criterion was adopted at this stage: prisoners were excluded if they did not provide the informed consent to be involved in the study.

**Supplementary material 2**. Comparison on PCL-R factor scores between SO and OO, and correlations between PCL-R factor scores in the two groups.

|  | Between group comparison | | | | | | Correlations |
| --- | --- | --- | --- | --- | --- | --- | --- |
| PCL-R factors | Groups | n | Mean | *SD* | Independent sample Student *t*-test | *p-value* | Pearson’s bivariate *r*-coefficients  (*p*-values) between  PCL-R factor scores |
| PCL-R F1 – Interpersonal and Affective Deficits | OO | 102 | 6.64 | 3.595 | -2.55_(188)_ | .011 | OO group: *r* = .495 (*p* = .000)  SO group: *r* = .462 (*p* = .000) |
|  | SO | 88 | 7.97 | 3.554 |  |  |  |
| PCL-R F2 - Antisocial Behavior | OO | 102 | 7.69 | 4.468 | 3.81_(188)_ | .000 |  |
|  | SO | 88 | 5.39 | 3.852 |  |  |  |

Note. PCL-R = Psychopathy Checklist-Revised, OO = Other offenders, SD = Standard Deviation, SO = Sex offenders.
